# Severe sleep apnea, Cheyne-Stokes respiration and desaturation in patients with decompensated heart failure at high altitude

**DOI:** 10.5935/1984-0063.20180028

**Published:** 2018

**Authors:** Leslie Vargas-Ramirez, Mauricio Gonzalez-Garcia, Camilo Franco-Reyes, Maria Angelica Bazurto-Zapata

**Affiliations:** 1 Fundacion Neumologica Colombiana, Sleep Center - Bogota - DC - Colômbia.; 2 Fundación Cardioinfantil-Instituto de Cardiología, Cardiology Deparment - Bogota - DC - Colômbia.; 3 Fundacion Neumologica Colombiana, Research Department - Bogota - DC - Colombia.

**Keywords:** Heart Failure, Sleep Apnea, Central Apnea, Cheyne-Stokes Respiration, Altitude

## Abstract

**Objectives:**

To determine the sleep-disordered breathing in patients with decompensated HF (DHF) at an altitude of 2640m.

**Methods:**

Polysomnogram during the first 48 hours of admission in patients hospitalized for DHF. Sleep apnea (SA) was defined as an apnea hypopnea index (AHI) > 5/hour and central sleep apnea (CSA) as central apnea index (CAI) ≥ 50% of the AHI.

**Results:**

Sixteen participants, LVEF 24.2±9.9%. All patients had SA, severe in 12 (75%), CSA in 8 (50%) and 7 (43.8%) presented Cheyne-Stokes respiration (CSR). Out of the eight patients with obstructive SA, five had a central component (CAI ≥ 5/h). The SpO2 decreased during sleep to 80.6±5.5% and in patients with CSR to 77.6±6.9%.

**Conclusions:**

At an altitude of 2640m all patients with DHF presented sleep apnea, most were severe, with CSA and a significant percentage of CSR that was associated with higher oxygen desaturation.

## INTRODUCTION

The estimated prevalence of heart failure (HF) in the European Union is 6.5 million, the prevalence reported by the European Society of Cardiology is 15 million people, and it has increased worldwide consistent with population aging, estimating that more than 23 million people have the disease^1^.

Sleep-disordered breathing (SDB) is frequent in patients with HF and the factors associated with it are instability in the ventilatory control, upper airway closure due to secondary edema by redistribution of fluid during supine position and obesity^2^. Sleep apnea (SA) prevalence in patients with a reduced ejection fraction is between 47 to 76%^3^, and in those with a preserved ejection fraction is 62%^4^. The presence of obstructive sleep apnea in these patients is an independent risk factor for mortality. Despite this, the SDB in patients with HF are not diagnosed or treated^3^^,^^5^.

The frequency of central sleep apneas in patients with heart failure and ventricular dysfunction ranges from 34 to 46%^6^. Variability can be explained by different factors such as age, gender, presence of hypocapnia or atrial fibrillation^7^. Additionally, it is associated with a lower ejection fraction, as well as a worse functional class^8^. In patients with stable severe heart failure, Cheyne-Stokes respiration (CSR) has been described in 30 to 50% of patients. The main mechanism is the fluctuation of PaCO_2_ above and below the apnea threshold; and in HF they is associated with low PaCO_2_ both in wakefulness and in sleep, prolonged circulation time and probably hypoxemia^9^^-^^11^.

Altitude confers an additional risk of central sleep apneas. At 2,000m, this type of respiratory events can appear in individuals without cardiac comorbidity, with a direct correlation between higher altitude and severity of central sleep apnea^12^. At the altitude of Bogota, the lower PaO_2_ and PaCO_2_ may come closer to the critical apnea threshold values could increase the presentation of CSR^13^.

It has been described that the diagnostic approach of sleep-disordered breathing (SDB) in patients hospitalized for decompensated heart failure (DHF) is a tool that allows the initiation of an adequate treatment in a shorter period of time, which may impact several outcomes^14^^,^^15^. Because the characteristics of sleep disorders in DHF at an altitude of 2640m are unknown, our objective was to describe the polysomnogram (PSG) findings in the first 48 hours of hospitalization of these patients.

## MATERIALS AND METHODS

### Patients

An analytical cross-sectional study was conducted in subjects older than 18 years old, living in Bogotá (altitude: 2,640m) hospitalized for DHF at the Fundación Cardioinfantil, Instituto de Cardiología. Patients with left ventricular ejection fraction < 45%, clinical signs of decompensated heart failure and elevated brain-natriuretic peptide (BNP) values were included. We excluded those with a previous diagnosis of sleep apnea or CPAP use, and those who required hospitalization in intensive care or permanent oxygen use. The research ethics committee approved the study and all subjects signed an informed consent.

### Polysomnography

Diagnostic type 2 polysomnography (PSG) were done within the first 48 hours of hospital admission for DHF, with Alice PDx (PhilipsRespironics, Murrysville, PA, USA), using montage as follows: two channels of electroencefalography (EEG) (C3, C4), electroculogram, submental and leg electromiogram, nasal airflow (P-TAF, Pro-Tech, Mukilteo, WA, USA), and thermistor (Respironics^®^), Respiratory inductance plethysmography (ZRIP), finger pulse oxymetry (Nonin^®^), electrocardiogram (DII), snoring and body position. Manual scoring of the study was done according to American Academy Sleep Medicine rules^16^. A minimum duration of sleep of 180 minutes was accepted.

### Definitions

Apnea was defined as a reduction of inspiratory airflow by ≥ 90% over 10 seconds, is obstructive if it meets apnea criteria and is associated with inspiratory effort, central if is associated with absent inspiratory effort and mixed if it meets apnea criteria and is associated with absent inspiratory effort in the initial portion of the event, followed by resumption of inspiratory effort in the second portion of the event; hypopnea was defined as a reduction of of inspiratory airflow by 30% from baseline lasting 10 seconds and accompanied by a 3% desaturation in oxygen. Cheyne-Stokes Breathing was scored when there were > 3 consecutive central apneas separated by a crescendo and decrescendo change in breathing amplitude with a central index > 5/h over > 2 hours of monitoring. Oxygen Desaturation Index (ODI) was the number of drops of SpO_2_ greater than 3% per hour of sleep^16^.

#### Statistical analysis

The distribution of the quantitative variables was evaluated using the Kolmogorov-Smirnoff test and they were presented as means and standard deviations or medians and interquartile ranges. Proportions were used for the qualitative variables. The clinical and PSG characteristics were compared among patients with central sleep apnea and obstructive apnea and among the groups with and without Cheyne-Stokes respiration. For continuous variables, Student’s t-test for independent samples or the Mann-Whitney U test was used and for the qualitative variables, the chi-square test or the Fisher’s exact test when the expected frequencies were less than 5. Hypotheses were formulated with two-tailed tests with a significance level of less than 0.05. The SPSS statistical software, version 10.0, was used.

## RESULTS

### Participants

We included 16 patients with decompensated heart failure, 75% men, aged 63.5±13.9. The left ventricular ejection fraction (LVEF) was 24.2±9.9%, 93.8% of the subjects were in NYHA class III-IV, 56.3% had ischemic heart disease and 43.8% had atrial fibrillation. The other demographic, clinical and echocardiographic variables are shown in Table 1.

**Table 1:** Demographic and clinical characteristics.

	Total (N=16)	Obstructive Apnea (N=8)	Central Apnea (N=8)	*p*
Age, years	63.5±13.9	64.4±11.6	62.6±16.6	0.811
Men	12 (75.0)	5 (62.5)	7 (87.5)	0.569
BMI, kg/cm^2^	26.5±4.6	28.8±4.4	24.3±3.7	0.041
Neck circumference, cm	37.9±4.2	38.5±2.8	37.4±5.3	0.623
Snoring	10 (62.5)	7 (87.5)	3 (37.5)	0.119
Observed pauses	7 (43.8)	5 (62.5)	2 (25.0)	0.315
Epworth Scale	8.5±4.9	9.1±6.6	7.9±2.7	0.632
Cause				
• Ischemic	9 (56.3)	6 (75.0)	3 (37.5)	0.315
• Dilated	4 (25.0)	1 (12.5)	3 (18.7)	0.569
• Other causes	3 (18.7)	2 (25.0)	1 (12.5)	0.999
Stage				
• C	13 (81.3)	6 (75.0)	7 (87.5)	
• D	3 (18.8)	2 (25.0)	1 (12.5)	0.999
Functional class				
• II	1 (6.3)	1 (12.5)	0 (0.0)	
• III	12 (75.0)	6 (75.0)	6 (75.0)	
• IV	3 (18.8)	1 (12.5)	2 (25.0)	0.513
LVEF, %	24.2±9.9	25.0±11.6	23.4±8.4	0.754
Pulmonary Hypertension	11 (68.8)	7 (87.5)	4 (50.0)	0.282
Diabetes	6 (37.5)	2 (25.0)	4 (50.0)	0.608
Systemic Hypertension	10 (62.5)	4 (50.0)	6 (75.0)	0.608
Atrial fibrillation	7 (43.8)	4 (50.0)	3 (37.5)	0.999
Chronic renal disease	4 (25.0)	3 (37.5)	1 (12.5)	0.569
Receiving				
• Diuretics	12 (75.0)	6 (75.0)	6 (75.0)	0.999
• Beta-bloquers	11 (68.8)	6 (75.0)	5 (62.5)	0.999
• ACE inhibitors	4 (25.0)	1 (12.5)	3 (37.5)	0.569
• Digital	2 (12.5)	2 (25.0)	0 (0.0)	0.467

Values as mean ± DE o N (%)

BMI: Body mass index; LVEF: Left ventricle ejection fraction; ACE: angiotensin converting enzyme.

*p*=differences between obstructive and central apnea

### Variables in the polysomnogram

In all subjects, sleep efficiency was low with a high arousal index. The 16 subjects had sleep apnea (AHI 45.5±21.6), 75% severe, 50% central sleep apnea (CAI > 50% of total AHI) and 7 (43.8%) Cheyne-Stokes respiration. Of the 8 patients with obstructive sleep apnea, 5 had a central component (CAI > 5). In patients with central SAHS, respiratory events occurred more frequently in non-REM sleep (*p*=0.005) and in supine (*p*=0.034). In all subjects with central and obstructive apnea, the desaturation index and the T_90_ were elevated, with a significant drop in oxygen saturation during respiratory events (Table 2).

**Table 2:** Polysomnogram characteristics.

	Total (N=16)	Obstructive Apnea (N=8)	Central Apnea (N=8)	*p*
Sleep efficiency, %	70.8±11.1	70.3±11.3	71.4±11.7	0.846
Arousal index/hour	32.7±16.1	31.5±16.1	34.0±17.2	0.763
AHI, number/hour	45.5±21.6	31.7±17.6	59.3±16.1	0.006
AHI REM, number/hour	31.8±30.8	22.2±34.7	41.5±24.9	0.223
AHI nREM, number/hora	47.1±24.9	31.0±19.6	63.2±18.8	0.005
AHI supine	46.3±26.8	32.5±27.3	60.1±19.0	0.034
AHI lateral	38.1±21.2	34.4±18.0	41.7±24.8	0.510
CAI, number/hour	25.9±22.7	7.4±8.9	44.4±15.7	<0.001
%CA	45.5±32.2	17.5±16.1	73.6±12.8	<0.001
CAI REM, number/hour (N=9)	7.4±10.0	0.3±0.5	11.0±10.7	0.057
CAI nREM, number/hour (N=9)	40.3±27.6	10.0±2.0	55.5±19.8	0.006
Cheyne-Stokes Respiration	7 (43.8)	1 (12.5%)	6 (75.0%)	0.041
OAI, number/hour	4.6±4.1	5.6±4.4	3.6±3.8	0.331
MAI, number/hour	3.6±3.4	3.3±3.4	4.0±3.6	0.727
HI, number/hour	11.4±7.1	15.4±7.4	7.4±4.0	0.018
T_90_, %	74.2±28.3	71.6±36.0	76.8±20.2	0.157
ODI	53.5±26.5	44.3±31.9	64.1±14.4	0.157
SpO_2_ wakefulness, %	87.8±4.1	87.3±5.5	88.4±2.3	0.601
SpO_2_ events, %	80.6±5.5	81.0±7.6	80.3±2.4	0.794

Values as mean ± DE o N (%)

AHI: Apnea hypopnea index; CAI: Central apnea index;OAI: Obstructive apnea index;

HI: Hypopneas index; SpO2: Oxygen saturation; ODI: oxygen desaturation index.

*p*=differences between obstructive and central apnea.

Subjects with obstructive apnea had a higher body mass index than patients in whom central sleep apnea predominated, without differences between these two groups in age, sleepiness scale, LVEF, type of heart disease, functional class, comorbidities or medications (Table 1).

### Cheyne-Stokes respiration

**Figure 1:**
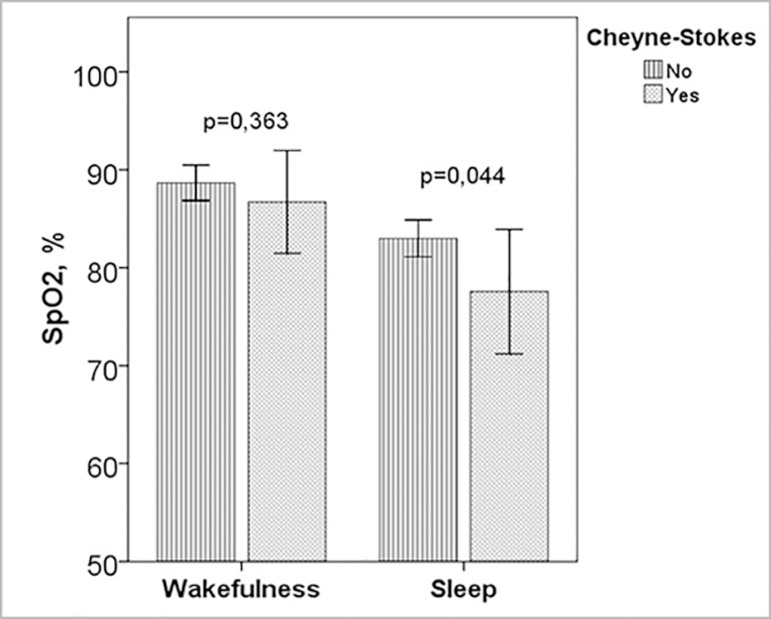
Oxygen saturation during wakefulness and sleep in patients with and without Cheyne-Stokes respiration Legenda: Subjects with Cheyne-Stokes had more desaturation during sleep respiratory events (*p*=0.044). SpO2: Oxygen saturation.

In patients with Cheyne-Stokes respiration AHI, CAI, desaturation index, T_90_ and desaturation during respiratory events were higher than in those without Cheyne-Stokes (Table 3) (Figure 1). Among these groups there were no differences in age, BMI, Epworth, antecedent of atrial fibrillation or LVEF.

**Table 3:** Patients with and without Cheyne-Stokes.

	Cheyne-Stokes (-) (N=9)	Cheyne-Stokes (+) (N=7)	*p*
Age, years	66.9±12.4	59.1±15.4	0.283
BMI, kg/m2	27.0±5.8	26.0±2.7	0.699
Neck circumference, cm	37.4±3.3	38.6±5.5	0.625
Epworth Scale	9.7±5.8	7.0±3.3	0.298
Atrial fibrillation	4 (44.4)	3 (42.9)	0.999
LVEF, %	25.0±10.9	23.1±9.1	0.722
Sleep efficiency, %	66.4±11.8	76.5±7.5	0.071
AHI, number/h	34.4±21.0	59.7±12.8	0.014
CAI, number/h	12.4±18.0	43.2±15.4	0.003
AHI REM, number/h	12.4±14.6	56.7±28.4	0.001
AHI NREM, number/h	36.2±26.9	61.1±13.5	0.043
AHI supine, number/h	44.3±27.0	48.8±28.5	0.754
AHI lateral, number/H	26.9±17.9	52.4±16.6	0.011
T_90_, %	71.3±33.1	77.9±22.9	0.672
ODI	39.3±20.5	74.9±19.4	0.006
SpO_2_ wakefulness, %	88.7±2.3	86.7±5.7	0.363
SpO_2_ events, %	83.0±2.4	77.6±6.9	0.044

Values as mean ± DE o N (%)

BMI: Body mass index; LVEF: Left ventricle ejection fraction; AHI: Apnea hyponea Index; AHI: Apnea hypopnea index;

CAI: Central apnea index; ODI: oxygen desaturation index;

SpO_2_: Oxygen saturation

*p*=differences between patients with and without CSR.

## DISCUSSION

The main finding of this study is that all patients included with DHF presented SA, most were severe, with the presence of central sleep apneas and with a significant percentage of Cheyne-Stokes respiration that was associated with higher desaturation.

Unlike other studies, all patients studied at the altitude had SA and in 75% of the cases it was severe. Sharma et al.^17^, in a recent publication, described sleep-disordered breathing in 91 of 105 hospitalized patients with decompensated heart failure, of whom only 40% had severe SAHS. In the work of Kauta et al.^14^, 78% of patients had AHI > 5/h.

The severity of SA in the patients included in this study may be explained by a lower ejection fraction (24.2±9.9) compared to the Sharma group (36.4±20.5) and Kauta (36.3±21.2), as well as a worse functional class and stage of heart failure (C and D), conditions that influence the pathophysiology of SA in these patients^3^^,^^18^. Unlike our study, in both aforementioned studies, the proportion of patients with predominantly central SA (CAI > 50% of total AHI) was significantly lower^17^. Similarly, the central sleep apnea index for the total group of patients with DHF (25.9±22.7) is markedly greater than that described in a previous study conducted by our group at the same altitude in subjects without heart failure, suggesting that the occurrence of central sleep apnea is not explained by altitude alone^19^.

In healthy individuals, the exposure to hypobaric hypoxia at high altitude causes an increase in the ventilatory drive triggering a pattern of alternating central sleep apneas and hypopneas with hyperventilation, oscillations of oxygen saturation and usually accompanied by arousals. In heart failure patients, abnormalities in CO_2_ chemoreceptor response have been demonstrated and may be the main determinant of the presence of Cheyne-Stokes, triggering respiratory events when PaCO_2_ falls below the apneic threshold^20^. Hypoxemia amplifies this response and it may explain the greater presence of Cheyne-Stokes respiration in these patients^12^^,^^21^.

It has also been shown that AHI is greater in patients with sleep apnea at 2,400m compared to those at lower altitudes (1,370m and at sea level), decreasing at the expense of fewer central events^22^. Oxygen saturation is significantly decreased at 1,860m and markedly at 2,590m^23^.

Patients described in this study had desaturation during wakefulness, with a significant decrease during respiratory events, with no differences between groups, which correlates with the severity of heart disease worsened by physiological changes related to altitude^24^. We do not know the impact that the elevated desaturation index and the severity of the desaturation may have in the evolution, control and prognosis of the disease^6^^,^^25^.

We found a significant number of patients with CSR in the upper range of what is described in the literature^11^. This could be related to the lower PaO_2_ and PaCO_2_ in the altitude of Bogota which are closer to the critical apnea threshold values^13^. Patients with CSR presented higher AHI (59.7±12.8) than those without CRS (34.4±21.0) and severity was not modified with the lateral decubitus position as described by Szollosi et al.^26^. Although T_90_ was similar among those who presented CSR and those who did not, we found a higher desaturation index and more severe desaturation during the respiratory events in CRS, in contrast to what was found in some reports where both PaO_2_ in wakefulness and SpO_2_ during sleep were normal and practically identical in patients with HF with and without CSR^11^.

There is some evidence that the presence of CSR is a marker of poor prognosis, with some contradictory data. However, it appears to have some deleterious impact, mediated by peaks in systemic blood pressure and heart rate, and by arousals related to increased sympathetic activity^27^.

The beta-blocker use rate was 68.8% in the total group, with no difference between the subgroups (obstructive and central) which has previously been described as a factor that does not influence the decrease in the proportion of central events^28^. Although atrial fibrillation has been described as a predictor of Cheyne-Stokes^29^, in this group of patients there was no difference in the antecedent of atrial fibrillation between those with and without CSR (42.9% *vs.* 44.4%, *p*=0.99).

Some limitations of the study are the small sample size and the lack of follow-up of patient outcomes. However, there was a careful selection of patients using clinical and laboratory criteria to classify them as DHF; only subjects living at high altitude were included and they were evaluated in the first 48 hours of hospitalization with a type 2 polysomnographic study without supplemental oxygen. To our knowledge, this is the first study performed at the altitude, describing respiratory disorders during sleep in patients with decompensated heart failure, which increases the knowledge of these pathologies at altitude. Further studies are required with a larger sample of subjects to assess the impact of SDB and treatment received.

## CONCLUSION

At an altitude of 2,640m, all patients studied with DHF presented sleep apnea, most were severe, with the presence of central sleep apneas and with a significant percentage of Cheyne-Stokes respiration that was associated with higher desaturation.
